# Species-Specific Chitin-Binding Module 18 Expansion in the Amphibian Pathogen *Batrachochytrium dendrobatidis*

**DOI:** 10.1128/mBio.00150-12

**Published:** 2012-06-19

**Authors:** John Abramyan, Jason E. Stajich

**Affiliations:** Department of Plant Pathology and Microbiology, University of California, Riverside, California, USA

## Abstract

*Batrachochytrium dendrobatidis* is the causative agent of chytridiomycosis, which is considered one of the driving forces behind the worldwide decline in populations of amphibians. As a member of the phylum *Chytridiomycota*, *B. dendrobatidis* has diverged significantly to emerge as the only pathogen of adult vertebrates. Such shifts in lifestyle are generally accompanied by various degrees of genomic modifications, yet neither its mode of pathogenicity nor any factors associated with it have ever been identified. Presented here is the identification and characterization of a unique expansion of the carbohydrate-binding module family 18 (CBM18), specific to *B. dendrobatidis*. CBM (chitin-binding module) expansions have been likened to the evolution of pathogenicity in a variety of fungus species, making this expanded group a prime candidate for the identification of potential pathogenicity factors. Furthermore, the CBM18 expansions are confined to three categories of genes, each having been previously implicated in host-pathogen interactions. These correlations highlight this specific domain expansion as a potential key player in the mode of pathogenicity in this unique fungus. The expansion of CBM18 in *B. dendrobatidis* is exceptional in its size and diversity compared to other pathogenic species of fungi, making this genomic feature unique in an evolutionary context as well as in pathogenicity.

## IMPORTANCE

Amphibian populations are declining worldwide at an unprecedented rate. Although various factors are thought to contribute to this phenomenon, chytridiomycosis has been identified as one of the leading causes. This deadly fungal disease is cause by *Batrachochytrium dendrobatidis*, a chytrid fungus species unique in its pathogenicity and, furthermore, its specificity to amphibians. Despite more than two decades of research, the biology of this fungus species and its deadly interaction with amphibians had been notoriously difficult to unravel. Due to the alarming rate of worldwide spread and associated decline in amphibian populations, it is imperative to incorporate novel genomic and genetic techniques into the study of this species. In this study, we present the first reported potential pathogenicity factors in *B. dendrobatidis*. *In silico* studies such as this allow us to identify putative targets for more specific molecular analyses, furthering our hope for the control of this pathogen.

## Introduction

Global decline in biodiversity is edging towards prehistoric proportions (1). This grim fact is epitomized by the recent catastrophic decline in amphibians, of which ~40% are imperiled (2). Various causes for this phenomenon are suspected, including climate change and habitat loss (3), but *Batrachochytrium dendrobatidis*, the causative agent of chytridiomycosis, is often cited as the most robust explanation (2). *B. dendrobatidis* is a pathogenic fungus of the phylum *Chytridiomycota* (4), a group comprised of generalist saprobes and parasites of various organisms ranging from algae to microscopic animals (5). Of the two pathogenic *Chytridiomycete* species identified to be vertebrate specific, *B. dendrobatidis* is the only one specific to amphibians (6, 7). This specificity has evolved despite the complex antimicrobial skin secretions which amphibians are known for (8). In addition to causing individual mortality, chytridiomycosis devastates entire populations, causing declines leading to eventual extinction (2).

From its discovery in 1998 until 2009, the physiological effect of *B. dendrobatidis* on the amphibian host was unknown (9). Recently, in a series of studies, Voyles and colleagues found that *B. dendrobatidis* causes electrolyte imbalance in the amphibian system (10-12). Additionally, *B. dendrobatidis*-infected frogs show a weak immune response to the pathogen. When transcripts of amphibian immunity-related genes were measured, the few genes that did show up-regulation did so at later time points of infection, contrary to common belief, indicating possible suppression of the immune system by *B. dendrobatidis* (13).

To date, the majority of studies involving *B. dendrobatidis* have focused on the ecology and the population-specific virulence of the fungus, providing little information for understanding its pathogenicity on a molecular level. However, the sequencing of genomes from two *B. dendrobatidis* isolates (JEL423 and JAM81) has opened the gates to genetic analyses on various levels, ranging from population-level genomics to functional genetics (14). Since *B. dendrobatidis* is aquatic and likely specific to its amphibian host (a vertebrate with a biphasic lifestyle), substantial restructuring of its genome would likely have been required in order to adapt to this new environmental niche, overcome host defense factors, and acquire nutrients from this new source.

Pathogens are in a constant evolutionary arms race with the hosts’ coevolved defenses in order to effectively attack the host as well as obtain nutrients (15). This close interaction is the likely reason why virtually all pathogenic fungi are host specific (none attack all plants or animals) and, furthermore, tissue specific (16). Some of the key innovations associated with the evolution of a pathogenic lifestyle in fungi include production of a host-specific toxin (*Alternaria alternata* [17]), morphological restructuring (conversion from yeast to hyphal state) (*Candida albicans* [18]), expansion of protein-coding genes (*C. albicans* [19]), and specific gene loss associated with reduction of diet breadth and host range (*Metarhizium anisopliae* [20]). Recent studies of the phylum *Ascomycota* revealed a striking number of gene gains and losses, numbering in the thousands for some species, in lineages where pathogenic lifestyles have evolved (21, 22).

One of the more commonly observed genomic modifications is lineage-specific expansion (LSE) of gene families and domains within a gene. These expansions often occur through tandem duplication events (23). In eukaryotes, nearly half of all paralogous protein clusters have been generated through LSEs (24). A recent study of the *Coprinus cinereus* genome revealed an overrepresentation of paralogous, multicopy genes in genomic regions with high rates of meiotic recombination, describing a possible mechanism for LSE generation in fungi (25). Due to the redundancy of the duplicates, gene and domain expansions provide raw material for the evolution of novel functions (26). New functional roles can arise through domain accretion or reshuffling (27, 28). Additionally, the availability of genomic material through LSEs also allows for a more effective response to new and diverse environmental pressures (24).

Domain expansions, which result in multimodularity, are classified as “homogenous” and “heterogeneous” (29). Homogenous expansions occur when domains are more closely related to each other than to a domain on another gene, likely being formed by tandem duplication events. Homogenous multimodularity is likely to lead to stronger binding affinity, since the overall affinity of the CBMs (chitin-binding modules) operating in tandem is greater than that of the individual domains working independently (29). Heterogeneous domain expansions, on the other hand, are observed when one of the domains is more closely related to a domain on another locus than to any domain in the same gene. Heterogeneous multimodularity allows for distinct ligand-binding specificities, potentially giving an enzyme multiple roles (29). Each type of domain expansion enhances protein function in different ways that may play specific roles in recognition of or adhesion to external chitin molecules.

While LSEs allow for the attainment of the genetic profile required for pathogenicity, they do not necessarily lead directly to pathogenesis but provide a foundation which increases the likelihood of development towards a pathogenic lifestyle. This pattern has been observed for keratinase and peptidase gene families in the *Onygenales* fungi and implicated in the evolution of *Coccidioides* as a vertebrate pathogen (30). Many LSEs found in fungi have pathogenicity-related functions, such as secondary metabolite production, melanin biosynthesis, carbohydrate binding, and cell wall degradation (24, 31). We observed that carbohydrate-binding module family 18 (CBM18 [chitin-binding module 18]; Pfam ID PF00187) is an LSE in *B. dendrobatidis* and sought to test whether its recent expansion could be predicted as a pathogenesis factor.

CBM18 is a subclass of chitin recognition domains that have evolved convergently in fungi, plants, and arthropods (32). The stereotypical chitin-binding domain is the hevein domain, first discovered in the latex of rubber trees (*Hevea brasiliensis*) (33). Most CBMs have 30 to 43 residues organized around a conserved four-disulfide core, known as the hevein domain of a chitin-binding motif (32, 34). Although proteins such as chitinases, chitin-binding lectins, and other similar molecules contain hevein-like domains, their roles are radically different due to their subcellular localization, molecular sizes, and overall structure (35). Additionally, binding may not be relegated to chitin itself but may also extend to any complex glycoconjugates containing *N*-acetyl-d-glucosamine (GlcNAc, the monomeric unit of chitin) or *N-*acetylneuraminic acid (Neu5Ac) (34).

When the genome of *B. dendrobatidis* was initially analyzed (C. A. Cuomo, J. E. Stajich, and I. V. Grigoriev, unpublished data), the expansion of CBM18 was particularly apparent due to its extraordinary size and breadth. Although multiple CBM loci are common (29, 36), we focused on this expansion due to its potential as a unique cell wall and/or cell surface protein component which may contribute significantly to the pathogenic character of *B. dendrobatidis*. In the rice blast fungus, *Magnaportha oryzae*, there are 36 copies of a similar domain distributed among 21 predicted proteins (37). The expanded domain in *Magnaportha oryzae* has a cysteine pattern similar to those of the *B. dendrobatidis* CBMs and the AVR4 protein, which was previously shown to protect fungal cell walls from chitinases (38). To better characterize the history of the expansion of CBM18 in the *B. dendrobatidis* genome, we performed a phylogenetic analysis of the domains and their encoding loci. We detected both homogenous and heterogeneous expansions within a relatively large number of *B. dendrobatidis* genes. We also tested for positive selection both within loci and within the domains themselves, uncovering significant evidence of recent positive selection at individual sites, which is suggestive of an adaptive role for many CBM18 domains. 

## RESULTS

### Pfam search results.

**FIG 1:**
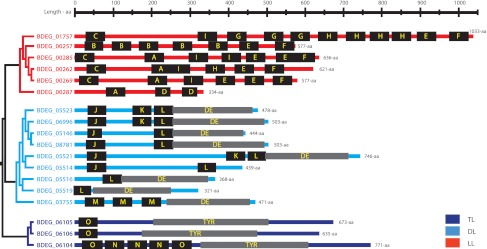
Domain positions on corresponding genes. CBM18 (chitin-binding module 18) domains are represented by black boxes with the phylogenetic domain groups labeled in yellow. Gray boxes represent non-CBM (chitin-binding module) domains identified on some genes. All domain lengths and positions are calculated on an amino acid level, beginning from the first residue of the gene. Widths of boxes and interdomain spaces are representative of relative domain lengths and spaces, respectively. Genes are color coded according to type. An unrooted cladogram generated from a Bayesian tree shows the phylogenetic relationships between genes. TL, tyrosinase-like; DL, deacetylase-like; LL, lectin-like; DE, deacetylase domain; TYR, tyrosinase domain; aa, amino acids.

When the CBM18 Pfam domain HMM was used to search against the predicted protein coding genes from the *B. dendrobatidis* genome, with an *E* value cutoff of 0.01, we identified 67 domain copies located on 18 genes (Fig. 1). The number of domains per locus ranged from a single domain to 11. The significance of this finding was furthered by our analysis of the closest sequenced genome of another chytridiomycete, *Homolaphlyctis polyrhiza. H. polyrhiza* has only 10 CBM18 copies. The *B. dendrobatidis* chitin-binding domain had a homologous (cysteine) pattern of X_3_CGX_7_CX_4_CCSX_4_CX_6_CX_3_C. This motif is also conserved between the *B. dendrobatidis* CBM18 and the hevein domain of plants (see Fig. S1 in the supplemental material). In addition to the CBMs, some of these genes also contained additional binding domains such as a tyrosinase domain (tyrosinase like [TL]) and a deacetylase domain (deacetylase-like [DL]) as identified using PHOG Universal Proteome Explorer, v.2.0. The third group consisted of genes with no secondary domains (lectin-like [LL]) (Fig. 1). Although other organisms have larger numbers of genes with CBMs, the 11 copies of CBM18 on BDEG_01757 likely represent one of the largest expansion within a gene (http://www.cazy.org/). Corresponding BDEG numbers also reflect the clustering of certain groups of genes with respect to chromosomal position (e.g., BDEG_06104 to BDEG_06106, BDEG_05514, BDEG_05516, BDEG_05519, BDEG_05521, and BDEG_05523), indicative of a tandem duplication pattern.

### Gene tree.

In order to understand the evolutionary relationship of the CBM18 genes, a Bayesian analysis was performed with the *H. polyrhiza* genes as a representative outgroup (see Fig. S2 in the supplemental material). Most major terminal clades in the tree show strong support with Bayesian posterior probabilities (BPP) ranging from 0.9 to 1.0. The tree is divided into three monophyletic and strongly supported clades consisting of TL (tyrosinase-like), DL, and LL genes. The clade representing the TL group is paraphyletic due to the presence of *H. polyrhiza* genes within the clade. The DL group is sister to the LL group, with a BPP of 1. Furthermore, intron-exon patterns and sizes display a distinct similarity within members of each group (see Fig. S3 in the supplemental material).

### Domain tree.

**FIG 2:**
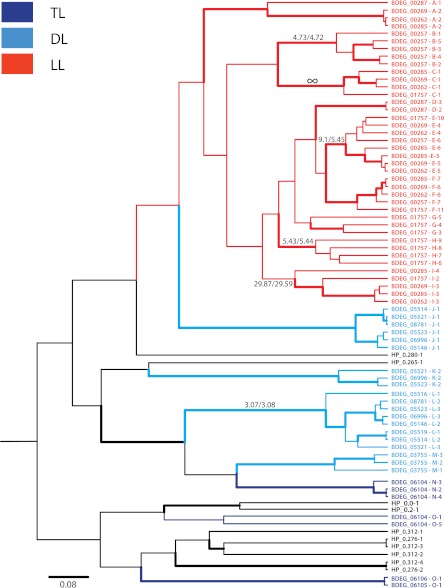
CBM18 domain phylogeny. Unrooted phylogram constructed using only domain sequences and implemented in BEAST v. 1.6.1 (65). *H. polyrhiza* domains were used as an outgroup and are represented in black. Domain names consist of the corresponding gene identifier, the phylogenetic group to which the domain belongs to, and the position in the gene which the specific domain holds. Thicker branches indicate Bayesian posterior probabilities of ≥0.80. ω values are given on branches which show positive selection. Branches are color coded according to gene type. TL, tyrosinase-like; DL, deacetylase-like; LL, lectin-like; HP, *H. polyrhiza*.

After the characterization of the CBM containing loci, the 67 individual domain sequences were isolated and aligned with the 10 from the outgroup *H. polyrhiza* (Fig. 2). This alignment was then used to build a phylogram using a Bayesian analysis in order to understand the relationship of the domains within and between loci. The Bayesian analysis revealed a relationship between domains that varies significantly from that of their parent loci. To simplify our analysis, we decided to partition the large number of domains further by assigning a letter to each monophyletic clade, referred to here as groups (Fig. 2).

Additionally, the individual domain groups were all monophyletic with respect to the type of gene they were located on (e.g., group N and O members are all found in various TL genes, while group J and L members are all found in DL genes). The domains found on the LL genes form a monophyletic clade, although with weak support, while the clade containing groups J, K, L, M, N, and O is comprised of members from both DL and TL genes (Fig. 2). Furthermore, group J, which is found in multiple DL genes, is monophyletic and sister to LL genes. Two *H. polyrhiza* domains were within the DL and LL clades, while the rest were paraphyletic with the TL group, in a similar manner to the gene analysis. Overall, the Bayesian analysis yielded a well-resolved phylogenetic tree which, although reflective of the evolutionary history of the loci, still shows marked individual evolutionary trajectories for the domains, independent of the type of gene they are located on.

### Domain order.

When the domain positions were mapped on the genes, we observed some general patterns which were reflective of the phylogenetic relationships of the domains. First, groups J, K, L, M, N, and O were restricted to the DL and TL genes, while the LL genes contained members of groups A, B, C, D, E, F, G, H, and I (Fig. 1). Furthermore, the members of each group occupied the same position in homologous genes (e.g., BDEG_06996, BDEG_05523, and BDEG_05521 contain domains from groups J, K, and L in the same order). There were some anomalous genes, however, such as locus BDEG_03755, which consisted entirely of domains from group M, which seems to be a sister group to the N group found in a TL gene (Fig. 2). Additionally, the DL genes mostly contained J, K, and L domains, with various losses of K and J observed. TL genes all contained group O domains, with one, BDEG_06104, containing three copies of N group domains, a unique domain found only in this locus. Interestingly, when the Bayesian analysis was performed on the domains, members of group O and N do not form a monophyletic clade (Fig. 2).

The LL genes also have unique patterns of gains and losses between them. In comparison to the DL and TL genes, the LL genes have a larger number of domains on average, ranging from 6 to 11. BDEG_00287 and BDEG_0285 in the LL clade, show a more distinct domain pattern than the rest (Fig. 1; also, see Fig. S2 in the supplemental material). The other four members of the LL clade (BDEG_00262, BDEG_00269, BDEG_00285, and BDEG_01757) show a similar pattern of the domain groups A, C, D, E, F, G, H, and I, with various duplications and losses throughout. BDEG_01757 stands out in all of the genes due to the 11 copies of the CBM18 domains it contains, including the unique G and H domains which, although related to the other LL domains, are unique (Fig. 1).

### Test for selection in domains.

Due to the disparities we saw between the domain phylogeny and the gene phylogeny, we decided to test for adaptive selection within domain groups. Convergent functional evolution may explain the paraphyletic groups which the DL and TL domains form (Fig. 2). Due to the short sequence lengths (30 to 43 residues), we decided to use the site models to identify specific amino acid sites within the domains potentially under positive selection. Although all of the domains contained characteristic CBMs, they showed a high degree of variability in their coding sequence. Therefore, analyzing the domains according to their monophyletic groups (A to O) gave more precise results than comparing all domains at once. We observed evidence for positive selection in 7 of the 14 groups analyzed.

The domains were analyzed under models M1 and M7 (null neutral models) and M2 and M8 (selection site models) (Table 1) as implemented in PAML 4.4. Of the 14 groups tested (excluding group D, which had only two members), we identified statistically significant adaptive selection in groups B, C, E, H, J, K, and L (Table 1; also, see Table S1 in the supplemental material). Group K was subsequently removed from the analysis due to an insufficient number of group members, which adversely affected the parameters of the analysis. The rejection of the neutral model in all cases was statistically significant, with *P* being <0.05 for group D and <0.005 for all other domain groups (Table 1; Table S1). In these groups, positively selected sites ranged from only 4.5% of the domains in group F to 37% of those in group J. Likewise, ω values ranged from a low of 3.07 in group L to ∞ (999.0) in group C, which is indicative of a *dS* value of ~0 (39). The selection models, M2 and M8, were in consensus with regard to the sites identified as showing strong adaptive selection, with M8 generally having a few additional sights coupled with stronger BEB support (see Table S1).

**TABLE 1:** Domain groups with adaptive selection signal

Comparison	Proportion (%)*^a^*	ω	2∆l	df	χ^2^
Group B					
M1 versus M2	45	4.73	5.27	2	0.036
M7 versus M8	22	4.72	5.39	2	0.034
Group C					
M1 versus M2	4.5	928.63	20.54	2	1.73 × 10^−5^
M7 versus M8	4.8	999.0	25.11	2	1.77 × 10^−6^
Group E					
M1 versus M2	12	9.10	15.46	2	0.00022
M7 versus M8	29	5.45	15.43	2	0.00022
Group H					
M1 versus M2	37	5.43	13.38	2	0.00062
M7 versus M8	37	5.44	13.46	2	0.00060
Group I					
M1 versus M2	5.2	29.87	10.03	1	0.00083
M7 versus M8	5.2	29.59	10.03	2	0.00331
Group L					
M1 versus M2	25	3.07	6.15	2	0.023
M7 versus M8	24	3.08	7.74	2	0.010

^a^ Percentage of sites with positive selections.

### Test for selection in whole genes.

From the previous analysis, we found that the majority of domain groups showing adaptive selection were from the LL clade. This finding, coupled with the fact that this clade contains the largest number of domain copies, led us to test for adaptive selection within the all LL genes. For this analysis, we decided to utilize the branch model as well as the branch site model in addition to the previously described site models, with the DL and TL clades as indicators of background evolutionary rate.

When the branch model was applied to the data set, the one-ratio model (null, neutral) was compared to the two-ratio model (selection). Using a chi-square test, we found that the two-ratio model fits significantly better than the one ratio model (see Table S2 in the supplemental material). The two-ratio model revealed that ω1f (foreground) was 0.81273, while ω0b (background) was 0.46233. Although the foreground LL clade had almost twice the ω value of the background, there was no indication of strong positive selection (see Table S2).

Given the difference in foreground and background ω scores, we decided to further our analysis by applying the branch-site model to isolate the specific sites which may be under positive selection. When model A was applied, and compared to the null neutral model M1 from the site models, we found a likelihood ratio statistic of 154.132 with a *P* value of <0.001, allowing us to reject the null neutral model M1. Of the sites, 22.7% showed significant positive selection, with a ω value of 4.612 for the foreground branches. Of the positively selected sites, >22% showed significant statistical support using BEB analysis (see Table S2 in the supplemental material). When model B was compared to M3, the neutral model (M3) was once again rejected with highly significant statistical support. Model B suggests moderate positive selection across all of the lineages (ω1b = 1.232) with strong adaptive selection in the LL clade, with a ω value of 5.0 at 20.6% of sites.

Lastly, the site models were used to analyze the entire genes, and models M1 and M2 were compared (see Table S2 in the supplemental material). The neutral model (M1) was rejected, although only 12.6% of sites were found to be under selection, with a ω value of 5.624 as identified by M2. Of this group, >15% showed high statistical significance using BEB. When models M7 and M8 were run, model M7 was once again rejected in favor of the selection model M8. Model M8 identified 16% of sites as showing positive selection with a ω value of 4.245; again >15% of these sites were statistically significant using BEB. Between models M2 and M8, model M8 had two additional sites, while the other six sites were shared between the two, with differing posterior probabilities.

## DISCUSSION

Of the more than 100,000 species of fungi described to date, only a very small proportion are pathogenic (40). Furthermore, many pathogens have nonpathogenic sister species, a pattern observed frequently in this kingdom. This pattern suggests that pathogenicity arose multiple times in the various fungal groups (15, 41). Due to the diverse genetic backgrounds that pathogens arise from, genomic modifications to attain pathogenicity are varied. However, one of the commonly identified elements that many fungi evolve en route to pathogenesis is an expansion of CBMs. Across the phylum *Ascomycota*, Soanes et al. (40) identified CBM18s (Pfam ID PF00187) in almost three times as many proteins (on average) in pathogenic lineages as in their nonpathogenic counterparts. Despite the association of CBMs and fungal pathogenesis, the exact roles of these domains are not known in fungi and are likely to be as diverse as the domains are among themselves.

The involvement of CBMs in pathogenesis is particularly intriguing in this study due to the pathogenicity-associated characteristics of the CBM18-containing genes themselves. The phylogenetic analysis of these genes revealed three well-supported clades: DL, TL, and LL. The TL group is also monophyletic with some of the *H. polyrhiza* genes, which is likely indicative of an ancestral association before the TL group gave rise to the LL and DL clades. The former two have secondary domains which have both been implicated as pathogenicity factors (genes essential for successful completion of the pathogenic life cycle but dispensable for saprophytic growth) (31, 40). The LL group contains only genes with CBM domains, which are themselves thought to be involved in pathogenesis. CBM domains do not necessarily function in catalytic activity, but they may be essential in chitin binding and substrate affinity for an enzyme (42). Furthermore, the LL group contains the largest fraction of the identified CBMs, both as a group and as individual genes. Through our analyses, we predicted that the members of the LL group function as lectins, which are commonly known to have multiple CBMs, as opposed to genes with a second type of functional domain, which are generally accompanied by only one CBM (34).

Although a role for the LL molecules in *B. dendrobatidis* has not been described, work with the tomato pathogen *Cladosporium fulvum* has shown that CBMs can bind to the chitin on the cell wall of a fungus, essentially functioning as a competitor of and limiting access for chitinases (38). A CBM-containing gene has been shown to protect fungi from basic plant chitinases through this mechanism (38). It is not unlikely that *B. dendrobatidis* has evolved this CBM expansion to function in defense of its cell wall in a manner similar to that of *Cladosporium*. Alternatively, *B. dendrobatidis* may utilize a chitin-binding ability to bind to nonhost chitinous structures (e.g., discarded insect integuments) in an as-yet-undescribed aspect of its ecology, an association chytrids are known for (5). Multiple CBMs can function synergistically to enhance binding affinity of a lectin for chitin (29, 36). The potential ability of *B. dendrobatidis* to survive within chitinous structures in the environment, without amphibian hosts being present, could have major implications for conservation and eradication efforts.

Such long repeats as we have observed in LL loci are common in proteins that are involved in protein-protein interactions and likely occur through tandem duplications (23, 43). One such example is the cytochrome P450 gene, which has been observed to have 40 to 140 copies in filamentous ascomycetes and basidiomycetes, compared to yeast, which has three (40, 25). However, the 11 copies of CBM18 in BDEG_01757 constitute the largest CBM18 expansion by far of any organism that we are aware of and may have functional properties that will be important to characterize in follow-up molecular genetic explorations. Alternative search approaches, as well as reanalysis with higher *E* values, are likely to discover more CBM18 domains as well as domain-carrying genes. However, these analyses would simply further support our conclusions of an expansion of this group of domains in this species.

In addition to their domain abundance and diversity, the LL clade also appears to show the strongest signal of adaptive evolution of the three groups. When we tested the domain groups for selection, 5 of the 6 groups identified as being under positive selection were from LL loci. Furthermore, when a *dN*/*dS* analysis was performed on the whole gene sequence, using the selection levels of DL and TL genes as background rates, LL genes showed a significantly stronger signal for adaptive evolution.

Anisimova et al. (44) discuss the effects of sequence length as well as sample numbers with respect to the power and effectiveness of the likelihood method to detect positive selection in the CBM18 domains. In their analyses, short sequences were more likely to produce a 2∆l value of 0, similar to sequences with low divergence. Sequence length may also have an effect on the χ^2^ approximations; however, Anisimova and colleagues (44) also showed that sequences as short as 50 codons show a normal χ^2^ distribution pattern in computer simulations. Our sequences are somewhat shorter than these averaging ~41 codons. However, none of the clades we report in this study as having positive selection display any of the aforementioned effects of short sequences.

Furthermore, Anisimova et al. (44) found that the number of taxa did not affect the shape of the 2**∆**l distribution in their simulation studies, while they used as few as five taxa. However, the number of taxa did negatively affect the power of the LRT, which may have had an effect on our analysis of clade K, which has only three members and thus was not reported as a significant result. Furthermore, Yang (45) suggests a minimum of four to five sequences. While some of our clades have less than this recommended number of sequences, most do not. All other clades reported in our analyses meet this criterion.

Additionally, de Jonge and Thomma (46) suggest that CBM-rich genes may be involved in repression of immune response by sequestering any chitin oligosaccharide breakdown products which may be released during invasion, thereby concealing the presence of the pathogen from the host. This may explain the lack of immune response observed by Rosenblum et al. (13).

While the LL loci are CBM rich and appear to be evolving at a higher rate than other loci, the DL and TL loci also reveal an interesting link to pathogenicity. The TL loci encode tyrosinase/catechol oxidase-like domains in addition to CBM18. Genes with putative tyrosinase activity are typically expanded in copy number among pathogenic fungal lineages and are associated with melanin biosynthesis (31). In fungi, melanins are involved in virulence by defending the cell against UV radiation, free radicals, and hydrolytic enzymes which can attack the cell wall and cellular components (47, 48). Melanized cell walls also show an electron-dense outer layer compared to albino mutants in *Wangiella dermatitidis* (49). Melanins also bind metals and function as redox-buffers, acting as a sink for unpaired electrons (50, 51). If such an electron-dense outer layer is formed in *B. dendrobatidis*, it may participate in electron transport disruption, which is thought to be the main cause of host amphibian death from *B. dendrobatidis* infections (12).

Interestingly, CBM18 domains in the TL group showed no statistically significant positive selection, indicating that these loci are most likely under purifying selection, possibly due to a functional link with the tyrosinase domain. However, there does seem to be some indication of convergent evolution, since the domains from group N, which likely arose from a tandem duplication of group O domains on a TL gene, form a well supported monophyletic clade with group M from the DL clade. The aforementioned scenario results in a TL gene with multiple, distinct domains. A recent study identified three chitin-binding sites in the locus of the archaeon *Thermococcus kodakaraensis* KOD1, where one domain had 1/3 the binding force of the other (52). The diversification in CBM function in this gene may fine-tune this TL gene to a multivalent substrate (29).

The DL clade is comprised of eight genes containing a deacetylase domain, which can be found to play a role in pathogenic lineages. Chitin deacetylases convert chitin into chitosan by a de-N-acetylation process, either changing the chitin polymers of the cell wall directly or providing a chitosan mask on top of it (53, 54). This process is significant because chitosan is a poor substrate for standard antifungal hydrolases such as chitinases and β-1,3-glucanases, which primarily target the outermost layers of the fungal cell wall (53). There is sparse literature on skin-secreted chitinases in amphibians, but amphibians do produce chitinases in the digestive system and pancreas and may form a symbiotic relationship with bacteria that produce these enzymes on their skin in order to fight off fungal infection (55, 56). Additionally, amphibian skin secretions have been shown to affect *B. dendrobatidis* survival directly (57). Of the 880 antimicrobial peptides identified in anurans to date, >20% are produced within special glands in their skin (58). Therefore, the expansion of a chitin deacetylase gene may represent evolved defensive measures that protect *B. dendrobatidis* from host-derived antimicrobial peptides which attack chitin (31).

In conclusion, we have identified a unique expansion of the CBM18 domain in *B. dendrobatidis* and evidence that positive selection has shaped the recent molecular evolution of these loci. Although these genes are putative with regard to their functional roles, their structures strongly indicate roles in pathogenesis and defense, two critical factors shaping genetic modifications in the *de novo* evolution of pathogenicity. Further molecular studies are required to show that the expression and function of these genes in the life cycle of *B. dendrobatidis* are relevant to defense, but bioinformatic analyses indicate a strong connection of the expansion of this gene family and the evolution of the pathogenic lifestyle of this unique fungus.

## MATERIALS AND METHODS

Predicted coding sequences (CDS) and their protein translations were obtained for *B. dendrobatidis* isolate JEL423 (accession no. AATT00000000) and *H. polyrhiza* isolate JEL142 (accession no. AFSM00000000) (http://fungalgenomes.org/public/Hp_JEL142/) (59). The CBM18 protein domain family (Chitin_bind_1) hidden Markov model (HMM) representation was downloaded from the Sanger Pfam24.0 database (60). The HMMER3 (61) application hmmsearch was used to perform a search of the CBM18 Pfam HMM against the *H. polyrhiza* and *B. dendrobatidis* protein databases with an expectation value (*E* value) of 0.01. The domains were identified and then extracted from the database using a custom Perl script utilizing BioPerl (62). Domain positions in the genes were identified manually.

To align the individual domains, the 67 *B. dendrobatidis* and 10 *H. polyrhiza* domain sequences identified by HMMER were extracted and aligned with T-Coffee (63). The resulting multiple protein sequence alignment was back translated based on the known CDS to create a codon alignment guided by the amino acid alignment using the BioPerl script (https://github.com/bioperl/bioperl-live/blob/master/scripts/utilities/bp_mrtrans.pl). This codon alignment was then used for molecular evolution and phylogenetic analyses of the domains. However, T-Coffee proved to be insufficient for the alignment of the entire locus due to variation in domain numbers, domain positions, and overall gene lengths. Instead, we used an anchored multiple alignment method implemented in the DIALIGN alignment tool (64) on the input CDS gene sequences (the coding regions are similar enough at the DNA level). This tool allows for user-specified anchor points to constrain the alignment to prioritize the alignment of the user-selected sequences (e.g., conserved domains) while the remaining parts of the sequence are aligned automatically. This method allowed us to create biologically meaningful alignments based on the *a priori* knowledge of homologous regions the alignment algorithms may not automatically recognize.

Phylogenetic analyses of the domain and gene CDS alignments were performed using a Bayesian Markov chain Monte Carlo (MCMC) analysis implemented in BEAST v. 1.6.1 (65). The program uses an MCMC method to sample tree and parameter space and can be summarized to generate a consensus tree with posterior probabilities for each clade representing confidence in the presence of the clade from the sampled phylogenies. The analysis was run with a minimum of 10 million generations and sampling every 100th tree. Subsequently, a consensus tree was generated using TreeAnnotator v.1.6.1 (65) and viewed using FigTree v. 1.3.1 (http://tree.bio.ed.ac.uk/software/figtree/). Additionally, the PHOG (PhyloFacts Orthology Group) Universal Proteome Explorer v.2.0 was used to annotate functionally and structurally important regions in the protein sequences in order to predict functional roles for proteins (66).

### Test for selection.

To test for selection both within lineages as well as within specific amino acid sites, we compared rates of synonymous (silent; *dS*) and nonsynonymous (amino acid replacement; *dN*) substitutions in the CBM18 domains. The *dN*/*dS* ratio (ω) measures selective pressure on amino acids. Thus, if *dN* significantly exceeds *dS*, divergence is likely to have occurred through positive selection. In other words, under selectively neutral conditions or even purifying selection, ω is less than or equal to 1, whereas ω will exceed 1 if an amino acid change is selectively advantageous. Finding that ω is significantly greater than 1 shows convincing evidence of adaptive selection (67).

To test for positive selection, we use a tree-based likelihood approach as described by Yang (68). Bayesian phylogenetic trees were constructed, as previously described, for each data set. Codon alignment files as well as their corresponding Bayesian tree files were submitted to the CODEML program in PAML v. 4.4 (45). CODEML was used to analyze the data under three different models and parameters.

The branch model is used to estimate positive selection along a specific lineage (54). Specifically, it allows the *dN*/*dS* ratio on a specified branch of the tree to differ from the average *dN*/*dS* ratio across the rest of the tree (two-ratio model). The null model fixes *dN*/*dS* at 1 on the specified branch. The one-ratio model is neutral and assumes the same ω ratio for all branches. The two-ratio model allows for two ω rates (69). Likelihood estimates assumed the codon substitution model of Goldman and Yang (70).

The site models allows the ω ratio to vary between codons (71). The null, neutral models do not allow for positive selection (ω ≤ 1) and are compared to an alternative hypothesis which does allow ω > 1. The first set of models consists of M1 and M2. M1 is a nearly neutral while M2 allows for selection, indicative of adaptive selection. Model M3 also allows for positive selection. Models M7 and M8 are two other models. M7 does not allow any positively selected sites (ω > 1), whereas M8 allows for an ω > 1 category of sites. M8 is less conservative and may identify positive selection in sites that the M2 analysis did not detect (72).

The branch-site model allows for the detection of positively selected sites on preselected lineages (45, 69). This model requires us to label specific clades or branches as a “foreground” group, which is being tested for selection in comparison to the “background” (all other branches in the tree). There are two models in the branch-site analysis: model A, which assumes that ω_0_ is 0 and ω_1_ is 1, and model B, which allows ω_0_ and ω_1_ to vary. Models A and B are identical to models M1 and M3, respectively, from the site model analysis (which is discussed above), except that the site models M1 and M3 assume ω_0_ and ω_1_ are the same across all branches of the phylogeny, since the site model does not use a labeled tree. If ω_2_ is >1, and if model A or B fits significantly better than model M1 or M3, then this is indicative of positive selection in the foreground branches. Confidence of specific evolutionary rate classes of sites is computed with a Bayes empirical Bayes (BEB) approach (73). Yang (45) describes these three models in greater detail. The likelihood estimates for each were compared using a hierarchical likelihood ratio test (hLRT) of twice the difference in the log likelihood value of the models being compared (2Δl), with the result approximating a chi square distribution.

## SUPPLEMENTAL MATERIAL

Figure S1 *B. dendrobatidis* CBM18 and hevein domain alignment. Cysteine residues are highlighted and numbered. Domain names consist of the corresponding gene identifier, the phylogenetic group to which the domain belongs, and the position in the gene which the domain holds. Lengths are represented in number of amino acid residues after each sequence. The three truncated domains, missing cysteine residues 6 and 7, are at the bottom of the list. Download FIG S1, DOCX file, 0.1 MB

Figure S2 Gene phylogram. Unrooted phylogram constructed using BEAST v. 1.6.1 (65) from alignment of entire gene sequences from CBM18-carrying genes. *H. polyrhiza* was used as an outgroup. All branches have Bayesian posterior probabilities of ≥0.90 with the exception of the one made with an asterisk. Branches are color coded according to gene type. TL, tyrosinase-like; DL, deacetylase-like; LL, lectin-like; HP, *H. polyrhiza*; *, BPP of 0.68. Download FIG S2, PDF file, 0.2 MB

Figure S3 Relative intron-exon positions in CBM18 genes. Introns are represented by yellow boxes along each gene. All domain lengths and positions are calculated on a base pair level, beginning from the start of the gene. Width of boxes and interintron spaces are representative of actual, relative intron lengths and spaces, respectively. Genes are color coded according to type. An unrooted cladogram generated from a Bayesian tree shows the phylogenetic relationships between genes. Download FIG S3, PDF file, 0.2 MB

Table S1 CODEML analysis of domain groups. TABLE S1, DOCX file, 0.1 MB.

Table S2 CODEML analysis of whole-gene alignment of putative lectins. TABLE S2, DOCX file, 0.1 MB.

Table S3 *dS* values of positively selected domain clades. TABLE S3, DOCX file, 0.1 MB.

Table S4 *dS* values of CBM18 genes. TABLE S4, DOCX file, 0.1 MB.
