# Supplementary material for: Species-Specific Chitin-Binding Module 18 Expansion in the Amphibian Pathogen Batrachochytrium dendrobatidis
Source: mBio. 2012 Jun 19;3(3):e00150-12. doi: 10.1128/mBio.00150-12 (PMC3569864; doi:10.1128/mBio.00150-12)
Supplement: Figure S1 — B. dendrobatidis CBM18 and hevein domain alignment. Cysteine residues are highlighted and numbered. Domain names consist of the corresponding gene identifier, the phylogenetic group to which the domain belongs, and the position in the gene which the domain holds. Lengths are represented in number of amino acid residues after each sequence. The three truncated domains, missing cysteine residues 6 and 7, are at the bottom of the list. Download [file mbo003121285sf01.docx]

1 2 34 5 6 7

**Hevein**  -EQCGRQAGGKLCPNNLCCSQWGWCGSTDEYCSPDHNCQSNCKD 43

BDEG_00287-A1 DGQCG-PEFKIKCFKNRCCSKKGYCGLGSYYCQ--AGCKKGYGD 41

BDEG_00269-A2 NGRCG-KKLNTKCDSNHCCSKDGECGSDEDYCE--AGCQKGYGR 41

BDEG_00262-A2 NGRCG-KKLNTKCDSNHCCSKDGECGSDEDYCE--AGCQKGYGR 41

BDEG_00285-A2 NGRCG-KKLNTKCDSNHCCSKDGECGSDEDYCE--AGCQKGYGR 41

BDEG_00257-B1 DGQCG-PNSG-ICPYNTCCSKYGFCGESSGHCG--VGCQKDYG- 39

BDEG_00257-B5 DGQCG-EGSGTRCPDRTCCSKYGFCGETSDHCG--VGCQKDYGI 41

BDEG_00257-B3 DGQCG-EGSGTRCPGKTCCSKYGFCGESSDHCG--VGCQKDYGI 41

BDEG_00257-B4 NSQCG-EEFGTRCPYNTCCSKYGFCGETSDHCG--VGCQKDYGI 41

BDEG_00257-B2 NSQCG-EEFGTRCPYNTCCSKYGFCGESSDHCG--NGCQKDYGI 41

BDEG_00262-C1 NGRCG-PHTGTTCPDNSCCSQSGRCGLSPSHCG--TGCQKPFGT 41

BDEG_00269-C1 DGRCG-PHTGTTCPDNSCCSQSGFCGLSPGYCG--TGCQKPFGT 41

BDEG_00285-C1 DGRCG-PNTG-ICPDNSCCSEFGFCGLSPNHCG--TGCRKPFGA 40

BDEG_01757-C1 DGQCG-PSSGTTCPNNSCCSESGQCGLSSEYCG--TGCQKPFGA 41

BDEG_01757-E10 DGRCG-EGFSTKCPGKTCCSGSGFCGKTPFHCG--VDCQKDYGS 41

BDEG_00269-E4 NNRCG-EGFNTQCPGKTCCSGKGFCGKTPSHCG--VDCQKGYGS 41

BDEG_00262-E4 DGRCG-EGFNTKCPGKECCSKHGFCGKTPSHCG--VDCQKGYGS 41

BDEG_00257-E6 DGQCG-EEFGTRCPDRTCCSGKGFCGKTPSHCG--IDCQKSYGS 41

BDEG_00285-E6 DGRCG-EGFNTQCQDKICCSGKGFCGKTPSHCG--DGCQKGYGS 41

BDEG_00285-E5 NNRCG-EGFNTQCQDKICCSKHGFCGKTPFHCG--VDCQKGYGS 41

BDEG_00269-E5 NNRCG-EGFNTQCQDKICCSKHGFCGKTPFHCG--DGCQKGYGS 41

BDEG_00262-E5 NNRCG-EGFNTQCQDKICCSKHGFCGKTPFHCG--DGCQKGYGS 41

BDEG_00285-F7 DGRCG-KEFKTKCPDRTCCSGSGFCGKTKSHCG--DGCQKDYGF 41

BDEG_00269-F6 DGRCG-KEFKTKCPDRTCCSGSGFCGKTKSHCG--DGCQKDYGF 41

BDEG_00262-F6 DGRCG-KEFKTKCPDRTCCSGSGFCGKTKSHCG--DGCQKDYGF 41

BDEG_00257-F7 DGRCG-KEFKTKCPGRTCCSGSGFCGKTKSHCG--DGCQKDYGF 41

BDEG_01757-F11 NNRCG-EGFNTKCPGRTCCSGSGFCGKTKSHCG--KGCQKNYGF 41

BDEG_01757-G5 DGQCG-KEFGTRCSDTLCCSRSGLCGDTDPHCG--KGCQEGYGI 41

BDEG_01757-G4 DNRCG-KVFGTKCPDTLCCSGDHFCGKTKGHCK--ENCQKDYGV 41

BDEG_01757-G3 NNRCG-KMFGTRCPDALCCSGNEFCGKTLFHCG--KDCQKDFGV 41

BDEG_01757-H9 DGRCG-EGFDTRCPGKECCSKSGFCGKTVGYCR--DGCQKDYGV 41

BDEG_01757-H8 DGLCG-DEVGTKCPGKDCCSKSGFCGKTVGYCG--KGCQESYGV 41

BDEG_01757-H7 NNQCG-WILGTQCSGKDCCSGNGFCGDTVGYCG--KGCQEGYGV 41

BDEG_01757-H6 DGLCG-KKFGLNCSGRDCCSKSGFCGESPGYCG--KGCQKDYGV 41

BDEG_00285-I4 DGQCG-EGFNTKCPNAQCCSGNGFCGSATGYCG--NGCQKDYGV 41

BDEG_01757-I2 DGQCG-SDINTKCPNGGCCSKNGVCGTTSGYCG--KNCQEGYGV 41

BDEG_00269-I3 NGQCG-SDLDTVCPDAQCCSKNGVCGETKGYCG--RECQRDYGV 41

BDEG_00285-I3 NGQCG-VDLDTVCPDAQCCSKNGVCGETKGYCG--RECQRDYGV 41

BDEG_00262-I3 NGQCG-VDLDTVCPGAQCCSKNGVCGTTKGYCG--RECQRDYGV 41

BDEG_05514-J1 NFGCG-PKAKTICPDNLCCSKYGACSRETSSCG--KNCQRGYGH 41

BDEG_05521-J1 NFGCG-PKAKTICPDNLCCSKYGACSRETSSCG--KNCQRGYGH 41

BDEG_08781-J1 NFGCG-PKAKTICPDNLCCSKYGACSRETSSCG--KNCQRGYGH 41

BDEG_05523-J1 --KCG-PKAKTICPDDLCCSEYGVCNRETDSCG--KNCQRGYGH 39

BDEG_06996-J1 --KCG-PKAKTICPDDLCCSEYGVCNRETDSCG--KNCQRGYGH 39

BDEG_05521-K2 NNQCG-LGYG-RCKGSLCCSEYGYCGSDDSYCV--AQCDPSYGE 40

BDEG_06996-K2 SDKCG-PKHG-RCKGSLCCSDRGYCGVDVSHCV--AQCDPLYGE 40

BDEG_05523-K2 SDKCG-PKHG-RCKGSLCCSDRGYCGVDVSHCI--SQCNPLYGE 40

BDEG_05516-L1 NNSCGRKYNKV-CPPESCCSRDNFCGTSTAHCG--IGCQGLFGI 41

BDEG_08781-L3 DNTCGRRYNKV-CPPESCCSRDGECGTTTAHCS--VGCQGLFGI 41

BDEG_05523-L3 DNTCGRRYNKV-CPPESCCSRDGECGTSTAHCS--VGCQGLFGI 41

BDEG_06996-L3 DNTCGRRVNKV-CPPESCCSRDGECGTTTAHCS--VGCQGLFGI 41

BDEG_05146-L2 DNTCGRQVNKV-CPPESCCSRDGECGTTTAHCS--VGCQGLFGI 41

BDEG_05519-L1 --MCGRKYNKM-CPPGSCCSRDGQCGTRSAHCG--IGCQGM--- 36

BDEG_05514-L2 DNMCGRKYNKM-CPPGSCCSRDGQCGTRSAHCG--IGCQGM--- 38

BDEG_05521-L3 DNTCGRQFNKM-CPPGSCCSHDNQCGTTTAHCG--IGCQNP--- 38

BDEG_03755-M3 DGTCGAKFGYQ-CDIGECCSQYGYCGVTSDYCS--VGCQNGFGT 41

BDEG_03755-M2 DGTCGAKFGYQ-CDIGECCSQYGYCGVTSDYCS--VGCQNGFGI 41

BDEG_03755-M1 DGSCGGSSGYR-CDTGECCSQYGYCGTTAAYCS--VGCQKSFGI 41

BDEG_06104-N3 DVKCGVGRGGK-CPNSLCCSQYGWCGNTDAHCG--IGCQNPFGT 41

BDEG_06104-N2 DVKCGVGRGGK-CPNSLCCSQYGWCGNTDAHCG--VGCQNPFGT 41

BDEG_06104-N4 DVKCGVGRGGK-CPNSLCCSQYGWCGNTDAHCG--VGCQNPFGT 41

BDEG_06104-O1 DPTCGSQHQNAHCPGTLCCSFVGYCNNSPVHCG--AGCQVLFGR 42

BDEG_06104-O5 NSRCGSQFQGLGCPDGLCCSAAGYCGITTAHCE--AGCQADSGI 42

BDEG_06105-O1 ASRCG-PGRG-VCPGRKCCSARGFCAITAKHCG--VGCQTPFGY 40

BDEG_06106-O1 ASRCG-PGRG-VCPGRKCCSARGFCAITVKHCG--VGCQTPFGY 40

BDEG_00287-D2 NNRCG-KKFGTRCPGQKCCSGKGFCGKTPSH------------- 30

BDEG_00287-D3 NNRCG-KKFGTRCPGQKCCSGKGFCGKTPSH------------- 30

BDEG_05146-J1 --KCG-PKAKTICPDDLCCSEYGVCNRETDS------------- 28
