# Supplementary figures and images for: Species-Specific Chitin-Binding Module 18 Expansion in the Amphibian Pathogen Batrachochytrium dendrobatidis
Source: mBio. 2012 Jun 19;3(3):e00150-12. doi: 10.1128/mBio.00150-12 (PMC3569864; doi:10.1128/mBio.00150-12)

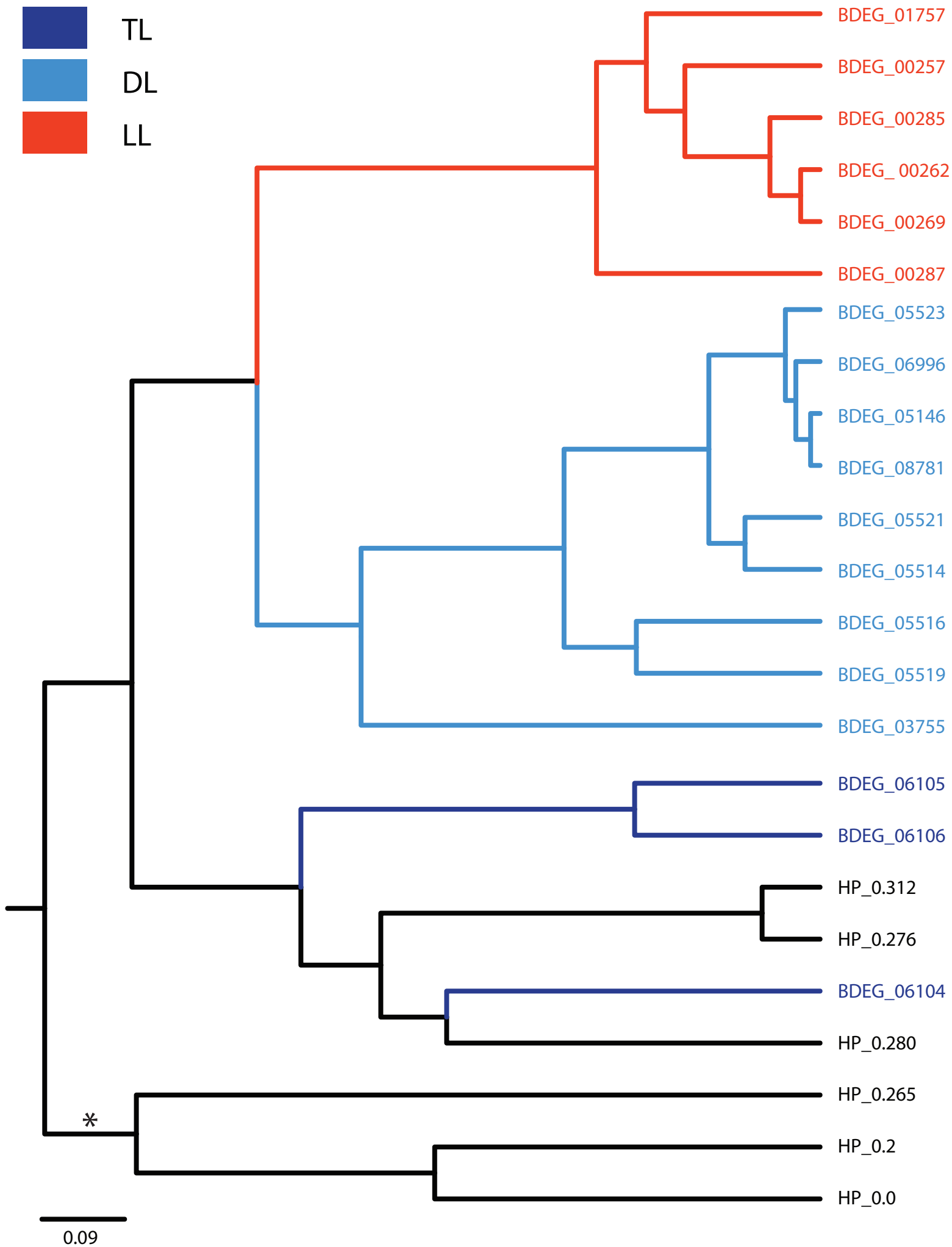

Supplement: Figure S2 — Gene phylogram. Unrooted phylogram constructed using BEAST v. 1.6.1 (65) from alignment of entire gene sequences from CBM18-carrying genes. H. polyrhiza was used as an outgroup. All branches have Bayesian posterior probabilities of ≥0.90 with the exception of the one made with an asterisk. Branches are color coded according to gene type. TL, tyrosinase-like; DL, deacetylase-like; LL, lectin-like; HP, H. polyrhiza; *, BPP of 0.68. Download [file mbo003121285sf02.pdf]

Length - bp

0 250 500 750 1000 1250 1500 1750 2000 2250 2500 2750 3000 3250 3500

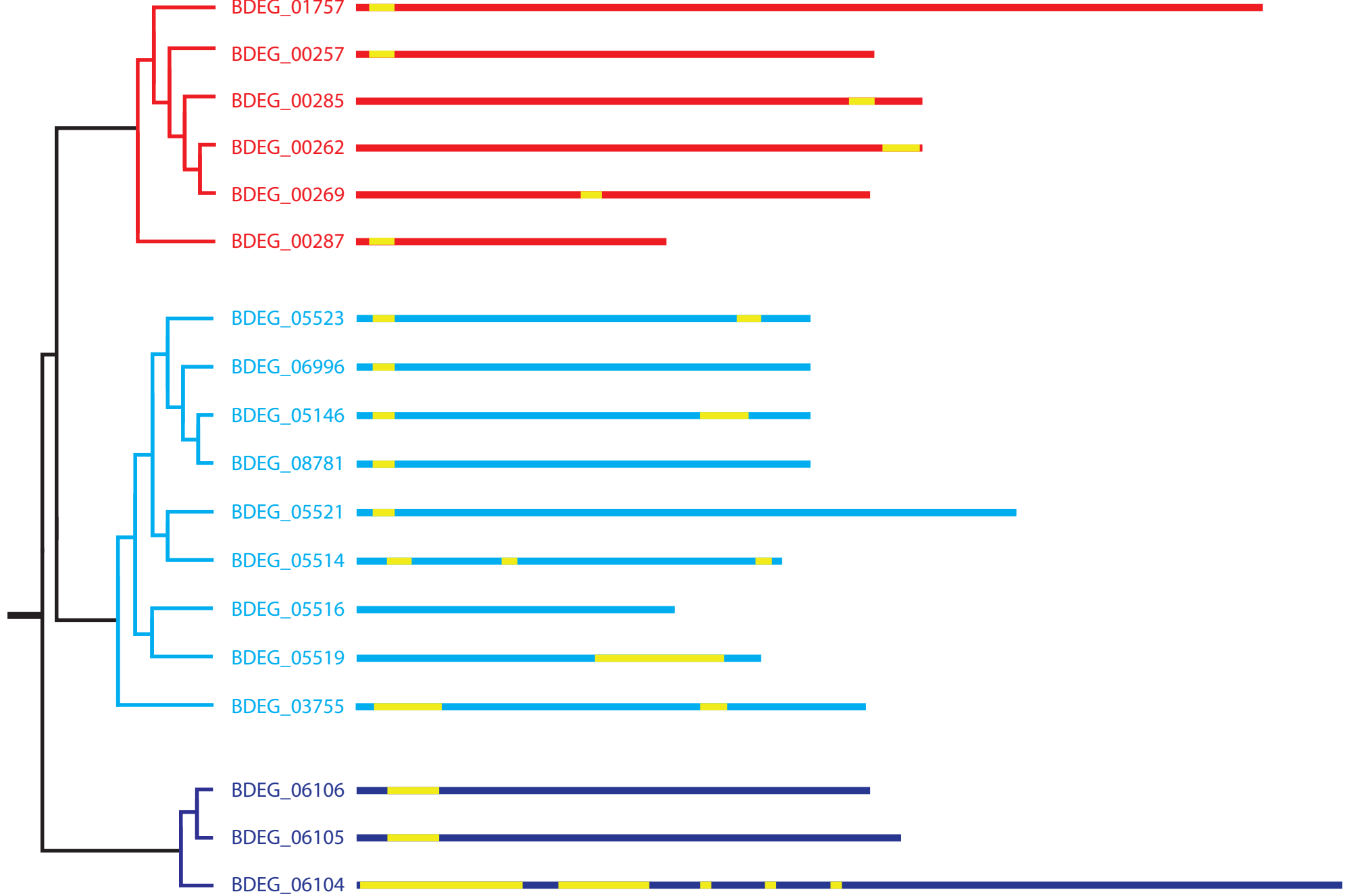

Supplement: Figure S3 — Relative intron-exon positions in CBM18 genes. Introns are represented by yellow boxes along each gene. All domain lengths and positions are calculated on a base pair level, beginning from the start of the gene. Width of boxes and interintron spaces are representative of actual, relative intron lengths and spaces, respectively. Genes are color coded according to type. An unrooted cladogram generated from a Bayesian tree shows the phylogenetic relationships between genes. Download [file mbo003121285sf03.pdf]
