# Supplementary material for: Species-Specific Chitin-Binding Module 18 Expansion in the Amphibian Pathogen Batrachochytrium dendrobatidis
Source: mBio. 2012 Jun 19;3(3):e00150-12. doi: 10.1128/mBio.00150-12 (PMC3569864; doi:10.1128/mBio.00150-12)
Supplement: Table S1 — CODEML analysis of domain groups. [file mbo003121285st1.docx]

**Table 1 - Codeml analysis of domain groups.**

**Group B**

**Model Paramenters** ωdN/dS l **2∆** l

M1 – neutral ω0 – 0.0, ω1 – 1.0 0.454 -302.493415 5.27*

p0 – 0.55 , p1 – 0.45 *(M1 vs. M2)*

M2 - selection ω0 – 0.17, ω1 – 1.0 1.1716 -299.859782

ω2- 4.73,

p0- .78, p1- 0.0, p2-.22

M7 – neutral p- 0.005, q- 0.005 0.5 -302.562681 5.39*

*(M7 vs. M8)*

M8 - selection p0 -0.78, p1 - .22 1.1714 -299.868598

p- 20.03, q- 99.0, ω- 4.72

M2-Positively Selected sites (BEB): 6,7,8,10,14,15,27,33

M8-Positively Selected sites (BEB): 6,7,8,10,14,15,27*,33

BEB= Bayes Empirical Bayes ; *: P < .05; **: P < .005.

**Group C**

**Model Paramenters** ωdN/dS l **2∆** l

M1 – neutral ω0 – 0.0, ω1 – 1.0 0.5988 -306.072536 20.54**

p0 - .40 , p1 - .60 *(M1 vs. M2)*

M2 - selection ω0 – 0.0, ω1 – 1.0 42.3022 -295.80258

ω2- 928.63,

p0- .24, p1- .71 , p2-.045

M7 – neutral p- 3.94, q- 0.005 1 -308.820728 25.11**

*(M7 vs. M8)*

M8 - selection p0 - .95, p1 – 0.048 48.7031 -296.267721

p- 6.6, q- 0.005, ω- 999.0

M2-Positively Selected sites (BEB): 7,10,23**,28,29*

M8-Positively Selected sites (BEB): 3,7,8,9,10,23**,28,29**,30

BEB= Bayes Empirical Bayes ; *: P < .05; **: P < .005.

**Group E**

**Model Paramenters** ωdN/dS l **2∆** l

M1 – neutral ω0 – 0.0, ω1 – 1.0 0.4998 -362.578452 15.46**

p0 - .50, p1 - .50 *(M1 vs. M2)*

M2 - selection ω0 – 0.0, ω1 – 1.0 1.8034 -354.848009

ω2- 9.10,

p0- .57, p1- .30, p2-.12

M7 – neutral p- .005, q-.005 0.5 -362.578446 15.43**

*(M7 vs. M8)*

M8 - selection p0 -.71, p1 -.29 1.8063 -354.861933

p-.005, q-.01, ω-5.46

M2-Positively Selected sites (BEB): 2*,9,14,16,20*,21**,29,33**,38

M8-Positively Selected sites (BEB):1,2**,3,9,11,14,16*,20*,21**,29,33**,34,38

BEB= Bayes Empirical Bayes ; *: P < .05; **: P < .005.

**Group H**

**Model Paramenters** ωdN/dS l **2∆** l

M1 – neutral ω0 – 0.02, ω1 – 1.0 0.6578 -370.533687 13.38**

p0 - .35, p1 - .65 *(M1 vs. M2)*

M2 - selection ω0 - .31, ω1 – 1.0 2.2068 -363.845437

ω2- 5.43,

p0-.63, p1- 0.0 , p2.37

M7 – neutral p- 0.11, q- 0.005 0.7 -370.578456 13.46**

*(M7 vs. M8)*

M8 - selection p0 - .63, p1 - .37 2.2083 -363.846255

p- 43.99, q-99, ω- 5.44

M2-Positively Selected sites (BEB): 3,6**,7**,8,10,11,13,26,28,33,37,38

M8-Positively Selected sites (BEB): 3*,6**,7**,8,10,11,13,26,28,33,37,38

BEB= Bayes Empirical Bayes ; *: P < .05; **: P < .005.

**Group I**

**Model Paramenters** ωdN/dS l **2∆** l

M1 – neutral ω0- 0.013, ω1- 1.0 0.5004 -360.933544 10.03**

p0 – 0.51, p1 – 0.49 *(M1 vs. M2)*

M2 - selection ω0 – 0.0, ω1 – 1.0 2.0536 -355.916367

ω2-29.87,

p0- .46, p1- .49 , p2-.052

M7 – neutral p- 0.006 , q-0.005 0.5048 -360.947206 10.03**

*(M7 vs. M8)*

M8 - selection p0 – 0.95, p1 – 0.052 2.0261 -355.929788

p- 0.005, q- 0.005, ω- 29.59

M2-Positively Selected sites (BEB): 6**,8,26**,28

M8-Positively Selected sites (BEB): 6**,8,11,26**,28,34

BEB= Bayes Empirical Bayes ; *: P < .05; **: P < .005.

**Group L**

**Model *p* Paramenters** ωdN/dS l **2∆** l

M1 – neutral 1 ω0 - .05, ω1 – 1.0 0.3564 -375.823808 6.15*

p0 - .68, p1 - .32 *(M1 vs. M2)*

M2 - selection 3 ω0 - .10, ω1 – 1.0 0.8299 -372.74817

ω2- 3.07,

p0- .76, p1- 0.0 , p2-0.25

M7 – neutral 2 p- .02, q- 0.02 0.4161 -376.636916 7.74*

*(M7 vs. M8)*

M8 – selection 4 p0 - .76, p1 - .24 0.8316 -372.76545

p- 11.5, q- 99.0, ω- 3.08

M2-Positively Selected sites (BEB): 3, 7, 8, 22, 23, 27, 38

M8-Positively Selected sites (BEB):3, 7*,8*, 11, 22, 23, 27, 38

BEB= Bayes Empirical Bayes ; *: P < .05; **: P < .005.
