# Supplementary material for: Species-Specific Chitin-Binding Module 18 Expansion in the Amphibian Pathogen Batrachochytrium dendrobatidis
Source: mBio. 2012 Jun 19;3(3):e00150-12. doi: 10.1128/mBio.00150-12 (PMC3569864; doi:10.1128/mBio.00150-12)
Supplement: Table S2 — CODEML analysis of whole-gene alignment of putative lectins. [file mbo003121285st2.docx]

**Table 2. Codeml analysis of Whole Gene alignment of putative lectins.**

**Model Parameters** ω **dN/dS** l **2∆**l

**Branch Models**

One-ratio ω = .55307 -26373.5 35.046**

Two-ratio ω0b =.46233 -26356.0

ω1f =.81273

**Model Parameters** ω **dN/dS** l **2∆** l

**Branch-Site Models**

Model-A p0=0.365, p1=0.408 -25932.94 154.132** (p2+p3=**0.227**), ω0b=0.134, *(Model-A vs. M1)*

ω1b=1.0, ω0f=.134, ω1f=1.0

ω2f=4.612

Model-B p0=0.41, p1=0.384, -25929.64 116.980**

(p2+p3=**0.206)** *(Model-B vs. M3)*

ω0b=0.160, ω1b=1.232,

ω0f=.160, ω1f=1.232

ω2f=5.0

**Model Parameters** ω **dN/dS** l  **2∆** l

**Sites Models**

M1-neutral ω0 - 0.133 0.6266 -26010.00 187.019**

ω1 - 1.0 *(M1 vs. M2)*

p0 - 0.43

p1 - 0.57

M2-selection ω0 - 0.13 1.249 -25916.49

ω1 - 1.0

ω2 - 5.624

p0 - 0.384

p1 - 0.489

p2 - 0.126

M3 – discrete ω0 – .2 0.8539 -25988.13

(K=2) ω1 – 1.586

p0 - .528

p1 - .472

M7 - neutral p - .433 0.566 -26007.1 218.72**

q - .332 *(M7 vs. M8)*

M8-selection p0 - 0.839 1.1047 -25898.34

p1 - .161

p - .522

q - .521

ω - 4.245

*: P < .05; **: P < .005.
