# Supplementary material for: Species-Specific Chitin-Binding Module 18 Expansion in the Amphibian Pathogen Batrachochytrium dendrobatidis
Source: mBio. 2012 Jun 19;3(3):e00150-12. doi: 10.1128/mBio.00150-12 (PMC3569864; doi:10.1128/mBio.00150-12)
Supplement: Table S3 — dS values of positively selected domain clades. [file mbo003121285st3.docx]

Supp Table 3 – dS values of Positively Selected domain clades

**Clade B**

BDEG_00257.dom4_7

BDEG_00257.dom2_7 0.1186

BDEG_00257.dom5_7 0.1174 0.1183

BDEG_00257.dom1_7 0.3313 0.2226 0.3187

BDEG_00257.dom3_7 0.3681 0.2531 0.3563 0.0208

**Clade C**

BDEG_00269.dom1_6

BDEG_00262.dom4_6 0.0330

BDEG_00285.dom1_7 0.1502 0.1920

BDEG_01757.dom1_11 0.1504 0.1159 0.1354

**Clade E**

BDEG_00262.dom1_6

BDEG_00269.dom2_6 0.0406

BDEG_00285.dom7_7 0.1231 0.0798

BDEG_00285.dom2_7 0.0617 0.0200 0.1011

BDEG_00257.dom6_7 0.2732 0.2202 0.1188 0.1950

BDEG_00262.dom3_6 0.1920 0.1449 0.0563 0.1224 0.0373

BDEG_00269.dom4_6 0.1428 0.0992 0.0950 0.0782 0.0958 0.0364

BDEG_01757.dom11_11 0.1451 0.1007 0.1836 0.0795 0.1623 0.1167 0.0747

**Clade H**

BDEG_01757.dom10_11

BDEG_01757.dom9_11 0.2561

BDEG_01757.dom4_11 0.3080 0.2940

BDEG_01757.dom3_11 0.3693 0.5221 0.2575

**Clade I**

BDEG_00285.dom3_7

BDEG_00285.dom6_7 0.5214

BDEG_01757.dom7_11 0.4931 0.6057

BDEG_00262.dom2_6 0.0747 0.5272 0.3541

BDEG_00269.dom3_6 0.1177 0.5192 0.4248 0.0363

**Clade L**

BDEG_05521.dom2_3

BDEG_08781.dom1_2 0.1235

BDEG_05523.dom2_3 0.1690 0.0693

BDEG_06996.dom1_3 0.1221 0.0000 0.0686

BDEG_05146.dom1_2 0.1248 0.0701 0.0341 0.0695

BDEG_05519.dom1_1 0.1441 0.0613 0.1511 0.0606 0.1294

BDEG_05514.dom1_2 0.1399 0.0598 0.1471 0.0591 0.1260 0.0000

BDEG_05516.dom1_1 0.4388 0.5713 0.6450 0.5636 0.5427 0.2598 0.3090
