# Supplementary material for: Species-Specific Chitin-Binding Module 18 Expansion in the Amphibian Pathogen Batrachochytrium dendrobatidis
Source: mBio. 2012 Jun 19;3(3):e00150-12. doi: 10.1128/mBio.00150-12 (PMC3569864; doi:10.1128/mBio.00150-12)
Supplement: Table S4 — dS values of CBM18 genes. [file mbo003121285st4.docx]

Supp Table 4 – dS values of CBM18 genes

BDEG_06105

BDEG_06106 0.6456

BDEG_06104 -1 5.1809

BDEG_03755 1.7306 3.006 1.7918

BDEG_06996 2.5376 -1 1.9717 2.4597

BDEG_05523 2.1146 -1 2.3305 1.5818 0.0954

BDEG_05146 2.3835 3.5249 -1 2.1406 0.0843 0.0894

BDEG_08781 3.5542 -1 2.9107 2.24 0.1366 0.1659 0.0904

BDEG_05514 -1 -1 3.1679 1.5042 0.3506 0.4091 0.3795 0.2341

BDEG_05521 2.5783 -1 2.5307 2.2864 0.3047 0.3056 0.2993 0.2203 0.2345

BDEG_05516 2.2002 -1 2.1639 2.5337 0.5944 0.5617 0.6391 0.5218 1.143 0.5263

BDEG_05519 3.3704 -1 1.9291 -1 0.5509 0.5288 0.5937 0.513 0.3809 0.4912 0.3538

BDEG_00257 2.2475 2.583 1.5286 2.3128 3.0876 -1 -1 3.5892 -1 -1 -1 3.2698

BDEG_00285 1.8521 2.166 1.9281 -1 1.9115 2.3105 2.0756 1.698 2.2538 2.6044 2.1678 -1 0.3531

BDEG_00262 1.6264 1.4488 2.1125 4.0508 2.6918 2.415 2.2713 2.2451 2.0313 3.2069 1.2727 2.3862 0.3404 0.1348

BDEG_00269 1.5063 1.3228 2.1858 3.9829 -1 3.5205 3.245 3.1432 2.5328 -1 1.1817 2.96 0.3421 0.1522 0.0422

BDEG_01757 1.9248 2.0224 1.9955 1.1931 1.7399 1.6488 1.752 1.7907 1.5677 2.0702 2.4794 1.1034 0.4628 0.2888 0.1902 0.1973

BDEG_00287 1.1414 1.1445 1.9135 0.6887 2.6046 3.189 2.3809 2.1838 2.4227 1.8929 2.7701 1.29 1.0681 1.087 0.7591 0.7569 0.5933
